# Update on mesenchymal stem cell-based therapy in lupus and scleroderma

**DOI:** 10.1186/s13075-015-0819-7

**Published:** 2015-11-03

**Authors:** Audrey Cras, Dominique Farge, Thierry Carmoi, Jean-Jacques Lataillade, Dan Dan Wang, Lingyun Sun

**Affiliations:** Assistance Publique-Hôpitaux de Paris, Saint-Louis Hospital, Cell Therapy Unit, Cord blood Bank and CIC-BT501, 1 avenue Claude Vellefaux, 75010 Paris, France; INSERM UMRS 1140, Paris Descartes, Faculté de Pharmacie, 4 avenue de l’observatoire, 75004 Paris, France; Assistance Publique-Hôpitaux de Paris, Saint-Louis Hospital, Internal Medicine and Vascular Disease Unit, CIC-BT501, INSERM UMRS 1160, Paris 7 Diderot University, Sorbonne Paris Cité, 1 avenue Claude Vellefaux, 75010 Paris, France; Hôpital du Val de Grace, Internal Medecine Unit, 74 boulevard de Port Royal, 75005 Paris, France; Percy Military Hospital, Department of Research and Cell Therapy, 101 Avenue Henri Barbusse, 92140 Clamart, France; Department of Immunology, The affiliated Drum Tower Hospital of Nanjing University Medical School, 321 Zhong Shan Road, Nanjing, 210008 China

## Abstract

Current systemic therapies are rarely curative for patients with severe life-threatening forms of autoimmune diseases (ADs). During the past 15 years, autologous hematopoietic stem cell transplantation has been demonstrated to cure some patients with severe AD refractory to all other available therapies. As a consequence, ADs such as lupus and scleroderma have become an emerging indication for cell therapy. Multipotent mesenchymal stem cells (MSCs), isolated from bone marrow and other sites, display specific immunomodulation and anti-inflammatory properties and appear as ideal tools to treat such diseases. The present update aims at summarizing recent knowledge acquired in the field of MSC-based therapies for lupus and scleroderma.

## Introduction

Autoimmune diseases (ADs) are a group of heterogeneous conditions characterized by aberrant activation of the immune system with failure of the immune regulation to maintain adapted tolerance. They are traditionally classified as “organ-specific AD”, where the consequences of organ failure can be improved by a replacement opotherapy or an organ transplantation, and as “diffuse or systemic AD”, notably including systemic lupus erythematosus (SLE) and systemic sclerosis (SSc). However, progressive identification of the genetic background of each AD type [1] and elucidation of the mechanisms associated with self-directed tissue inflammation, unrelated to T- or B-cell abnormalities, revealed the important differences between autoimmunity and autoinflammation [2]. SLE, type 1 diabetes, and autoimmune thyroiditis are polygenic ADs with a predominant autoimmune component, whereas other polygenic ADs, such as Crohn’s disease, are characterized by a predominant autoinflammatory component. Therefore, the optimal treatment of AD should be discussed in light of this specific pathological continuum between autoimmunity and autoinflammation, which variably interacts in each AD phenotypic expression. Indeed, chronic immunosuppression is responsible for high treatment-related morbidity and still is associated with significant disease- and treatment-related mortality, notably in patients with severe inflammatory SLE or refractory SSc and with kidney, heart-lung, or brain damage. With a view to developing innovative therapies for AD, mesenchymal stem cell (MSC)-based therapies theoretically appear as ideal tools to target the respective autoinflammatory and autoimmune components of such diseases, and this update aims at summarizing recent knowledge acquired in the field.

## A need for innovative stem cell therapies in severe or refractory forms of systemic lupus erythematosus and systemic sclerosis

SLE, with a prevalence of 40 to 50 out of 100,000 people, is a heterogeneous chronic multisystemic autoimmune inflammatory disorder whose original flare can be controlled by conventional immunosuppressive therapy. However, definitive cure is rarely achieved by this therapy and life-long immunosuppression is often required. Response rates vary from 20 to 100 % at 6 months according to the definition of response or improvement, the extent of visceral damage, the ethnic origin, and the socioeconomic profile. First-line validated standard therapies used to induce remission within the first 6 to 9 months of disease flare are the corticosteroids in combination with either (a) cyclophosphamide (CY), using the classic National Institutes of Health regimen or lower doses for shorter duration over the course of 3 months with a similar efficacy, according to the Eurolupus regimen [3, 4], or (b) mycophenolate mofetil, with good efficacy and tolerability [5, 6]. Other monoclonal antibodies against the T- or B-cell receptors, such as rituximab as an anti-CD20, or against the adhesion molecules involved in the T- or B-cell interaction and their co-stimulatory signals, have been used despite the paucity of validated therapeutic targets and the failure to demonstrate the efficacy of rituximab in renal and extra-renal manifestations of SLE [7]. In 2011, a monoclonal antibody against B cell-activating factor of the tumor necrosis factor family (BAFF), belimumab anti-Blys, was the first targeted therapy to demonstrate its efficacy in mild to moderate SLE by a randomized clinical trial [8]. Despite early diagnosis and treatment with immunosuppressive agents as well as a tight control of hypertension and infections, there is still a subgroup of patients with SLE that does not respond to the treatment and that has 10-year mortality of 10 % [9]. In addition, early death from rapidly progressive atherosclerosis in SLE suggests that, despite apparent reasonable disease control, subclinical inflammatory disease promotes endothelial damage and plaque formation and that prolonged exposure to corticosteroids and immunosuppressive drugs leads to further damage beyond the SLE itself.

SSc, which has a prevalence of 5 to 50 per 100,000, is a rare AD characterized by early vascular endothelium damage with consequent activation of the immune response and enhanced collagen synthesis, leading to progressive fibrosis of the skin and internal organs. Both antigen stimulation and genetic susceptibility may contribute to autoimmunity, with consequent early T-cell infiltration as well as B-cell and fibroblast activation, by pro-fibrotic cytokines, mainly transforming growth factor-beta (TGF-β) and connective tissue growth factor. Most patients progress, and the overall 10-year survival is only 66 %, and there is significant morbidity and altered quality of life among survivors. In rapidly progressive SSc, mortality rates reach 30 to 50 % in the first 5 years after disease onset, according to the extent of skin, cardiopulmonary, and renal involvement [10]. No treatment has ever shown any benefit in this severe disease, except autologous hematopoietic stem cell transplantation (HSCT), whose efficacy was recently established by a unique international multi-center, open-label phase III, ASTIS (Autologous Stem cell Transplantation International Scleroderma) trial [11] that enrolled 156 patients over the course of 10 years with early diffuse cutaneous SSc, showing that HSCT confers a significantly better long-term survival rate than 12 monthly intravenous pulses of CY.

In this context, new therapeutic approaches with fewer long-term side effects are warranted. Bone marrow (BM) stromal cells or MSCs, which can also be obtained from other human tissues, have recently enlarged the therapeutic tool set for SLE and SSc. Because MSCs display specific immunomodulation and immunosuppressive properties as well as regenerative potential, there is a strong rationale for MSC-based therapy in SLE and SSc to treat their respective autoimmune and autoinflammatory components at a certain time point of each disease evolution.

## Biology of mesenchymal stem cells

### Definition, isolation, and characterization of mesenchymal stem cells

MSCs were originally identified in BM by Friedenstein in 1976 as a fibroblast-like cellular population capable of generating osteogenic precursors [12]. Since then, these cells have been extensively investigated and given various names, until 1991 when Caplan proposed the definition ‘mesenchymal stem cells’ (MSCs) [13], which after consensus of the Mesenchymal and Tissue Stem Cell Committee of International Society for Cellular Therapy (ISCT) was changed to “multipotent mesenchymal stromal cells”. ISCT has provided three minimal criteria to define MSCs [14]: (a) plastic adherence in standard culture conditions; (b) differentiation into osteoblasts, adipocytes, and chondroblasts under specific conditions in vitro; and (c) expression of nonspecific markers CD105, CD90, and CD73 along with the lack of expression of hematopoietic markers such as CD34, CD45, CD14 or CD11b, CD79a, or CD19. MSCs show intermediate levels of major histocompatibility complex (MHC) class I molecules on their cell surface and have no detectable levels of MHC class II, mainly HLA-DR, and co-stimulatory molecules (CD40, CD80, and CD86), which allow their transplantation across MHC barriers [15]. Therefore, their privileged immunological phenotype makes them an appropriate stem cell source for allogeneic transplantation. They can also synthesize trophic mediators, such as growth factors and cytokines—macrophage colony-stimulating factor, interleukin-6 (IL-6), IL-11, IL-15, stem cell factor, and vascular endothelial growth factor—involved in hematopoiesis regulation, cell signaling, and modulation of the immune response [16].

BM-MSCs were discovered first, and the BM was considered the main source of MSCs. BM-MSCs are classically expanded in vitro by consecutive passages in fibroblast growth factor-supplemented cell culture medium from the plastic-adherent BM cell population. Subsequently, MSCs, facilitated by their ability to adhere to plastic, have been isolated from various other sources such as skeletal muscle, adipose tissue, dental tissue, synovial membranes, placenta, cord blood, and Wharton’s jelly by using enzymatic tissue digestion and density gradient centrifugation methods [17]. These alternative sources are very attractive because BM harvesting is rather invasive and painful and is associated with potential donor-site morbidity. Moreover, because of the rarity of MSCs in the BM, where they represent 1 in 10,000 nucleated cells, tissues such as umbilical cord (UC) or adipose tissue (AT) represent promising sources. Indeed, MSCs can be more easily isolated from these tissues and considerably larger amounts of UC- or AT-derived MSCs can be obtained, compared with the BM. MSCs from these different sources share many biological features, although studies reported some differences in their immunophenotype, proliferative capacity, differentiation potential, or gene expression profile [18, 19].

### Immunomodulatory properties of mesenchymal stem cells: evidence from in vitro data

Compared with other stem cell sources, such as hematopoietic stem cells (HSCs), MSCs appear as a promising source for overcoming autoimmunity because of their immunosuppressive properties [20]. MSCs modulate the immunological activity of different cell populations as shown by in vitro data. Their most important effects are T-cell proliferation and dendritic cell (DC) differentiation inhibition, which are key activating factors of autoimmune disorders. MSCs are effective in inhibiting proliferation of CD4 and CD8 T cells as well as memory and naïve T cells [21]. This mechanism relies on both cell–cell contact and several specific mediators, produced by MSCs, such as TGF-β1, prostaglandin E_2_, and indoleamine 2,3 deoxygenase [22]. The ability to suppress T-cell responses to mitogenic and antigenic signals is explained by a complex mechanism of induction of “division arrest anergy”, responsible for maintaining T lymphocytes in a quiescent state. Thus, the MSCs trigger the inhibition of cyclin D2 expression, thus arresting cells in the G_0_/G_1_ phase of the cell cycle [23]. MSCs also inhibit the production of interferon-gamma (IFN-γ) and increase the production of IL-4 by T helper 2 cells. This indicates a shift in T cells from a pro-inflammatory state to an anti-inflammatory state [24, 25]. MSCs also stimulate the production of CD4^+^CD25^+^ regulatory T cells, which inhibit lymphocyte proliferation in allogeneic transplantation [26]. In addition, MSCs inhibit B-cell proliferation through arrest at the G_0_/G_1_ phase of the cell cycle and production of IgM, IgA, and IgG as well as their chemotactic abilities [27, 28]. A recent study demonstrated that this effect of MSCs on B cells is mediated by T cells [29]. However, some contradictory data showed that, in some culture conditions, IgG secretion and B-cell proliferation can be induced and B-cell survival sustained, and this effect does not depend on the presence of IFN-γ in the culture [30, 31]. MSCs have been demonstrated to interfere with DC differentiation, maturation, and function [32–34]. MSCs obtained from healthy human donors can indirectly reduce T-cell activation by inhibiting DC differentiation (mainly DC type I) from monocytes [35].

Although the majority of data dealing with the immunomodulatory effects of MSCs are derived from BM-MSCs, some of these effects have also been described for MSCs from other sources. Results from studies comparing the immunomodulatory effects of various tissue-derived MSCs are controversial. Some studies concluded that BM- and UC-MSCs show similar effects, whereas others demonstrated that UC-MSCs have a higher capacity of inhibiting T-cell proliferation than adult MSCs [36, 37]. Some studies also indicate that AT-MSCs can be more effective suppressors of immune response compared with BM-MSCs. Indeed, AT-MSCs modulate mitogen-stimulated B-cell immunoglobulin production in vitro to a much greater extent than BM-MSCs. Also, in comparison with BM-MSCs, they inhibit, significantly more, the differentiation of blood monocytes into DCs and the expression of functionally important co-stimulatory molecules on the surface of mature monocyte-derived DCs [38, 39]. It may be postulated that MSCs express a different set of molecules depending of their tissue of origin, resulting in different immunosuppressive activities. Taken together, these in vitro data demonstrate that MSCs modulate the action of the various cells that are involved in immune response and preferentially inhibit T-cell proliferation and differentiation of DCs. However, it would be important to further investigate the molecular mechanisms that underlie the immunomodulatory properties of various tissue-derived MSCs since these differences may have functional relevance to the therapeutic use of these cells.

## Mesenchymal stem cell-based therapy in animal models

Animal models of AD can be divided into two categories. The hereditary and spontaneous AD models, such as murine (BXSB) lupus, are characterized by autoimmune manifestations that affect the majority of the animals of a susceptible line and by a strong genetic predisposition displayed by the HSCs and manifested by anomalies of thymic development and/or function of lymphocytes B or T or antigen-presenting cells such as macrophages. Other experimental models, such as arthritis adjuvant and experimental acute encephalomyelitis [40], use active immunization by exposure to a foreign antigen to induce the AD. The rationale for using MSCs for the treatment of autoimmunity was first demonstrated in experimental acute encephalomyelitis, a model for multiple sclerosis [25]. Subsequently, several preclinical studies evaluating MSC injection in a collagen-induced arthritis model [41] or in an autoimmune type 1 diabetes model [42] provided support for the potential therapy of other ADs, including SLE and SSc.

### Animal models of systemic lupus erythematosus

Both Fas mutated MRL/lpr mice and NZB/W F1 mice are widely used as genetically prone lupus models, which demonstrate progressive nephritis, elevated serum autoimmune antibodies, and immune abnormalities. The role of BM-MSC transplantation in SLE and its efficacy compared with conventional CY treatment has been investigated in MRL/lpr mice as an SLE mouse model [43, 44]. MSC injection resulted in a significant reduction in serum levels of anti-double-stranded DNA (anti-dsDNA) antibodies IgG and IgM, ANA, and immunoglobulins IgG1, IgG2a, IgG2b, and IgM as well as an increased serum albumin level. When compared with MSCs, conventional CY treatment partially reduced the levels of serum autoantibodies and immunoglobulin IgG2a, restored albumin level, and failed to reduce circulating immunoglobulins IgG1, IgG2b, and IgM. MSC treatment improved renal disorders, specifically restoring kidney glomerular structure and reducing C3 and glomerular IgG deposition. Although CY treatment could reduce glomerular IgG deposition, it did not restore the glomerular structure and C3 accumulation. MSC treatment, but not CY treatment, was able to completely restore renal function, shown as normalization of serum and urine creatinine levels in MRL/lpr mice, in comparison with disease-free control mice. In their study, Ma et al. determined that murine BM-MSC transplantation improved nephritis in MRL/lpr mice by suppressing the excessive activation of B cells via inhibition of BAFF production [45]. Nevertheless, in a similar study conducted in a different SLE mouse model (NZB/W), systemic MSC administration did not provide any beneficial effect and in fact worsened the disease [46, 47]. To resolve these conflicting results, Gu et al. assessed the differential effects of allogeneic versus syngeneic MSC transplantation on lupus-like disease in both mice models [48]. They showed that, in MRL/lpr and NZB/W mice, both normal MSCs and lupus MSCs from young mice ameliorated SLE-like disease and reduced splenic T and B lymphocyte levels. However, lupus MSCs from older NZB/W mice did not significantly reduce spleen weights, glomerular IgG deposits, renal pathology, interstitial inflammation, or T or B lymphocyte levels. This study suggests that allogeneic MSCs may be preferred over syngeneic lupus-derived MSCs given the decreased overall effectiveness of post-lupus-derived MSCs, which is partly triggered by the disease and is not exclusively an intrinsic defect of the MSCs themselves. The same group reported that human lupus BM-MSCs are not as effective as human healthy BM-MSCs and umbilical cord-derived MSCs (UC-MSCs) in ameliorating disease in MRL/lpr mice [49]. Moreover, in vitro assessments of immunomodulatory functions detected a reduced capacity of lupus BM-MSCs to inhibit IFN-γ production and CD19^+^ B-cell proliferation, although inhibition of CD3^+^ proliferation and IFN-γ licensing results were indicative of immune activity by lupus BM-MSCs. Although these studies showed that lupus MSCs are not yet a suitable source of MSCs for cell therapy, it is important to continue to define differences in MSCs because it appears that donors and the origin of the MSCs impact their function.

Some studies evaluated the effectiveness of MSCs derived from sources other than BM. Sun’s team had showed that UC-MSCs alleviated lupus nephritis in MRL/lpr mice in a dose-dependent manner [50]. Both single and multiple treatments with UC-MSCs were able to decrease the levels of 24-h proteinuria, serum creatinine, anti-dsDNA antibodies, and the extent of renal injury, such as crescent formation. Further studies dealing with the underlying mechanisms showed that UC-MSC treatment inhibited renal expression of monocyte chemotactic protein 1 and high-mobility group box 1 expression but that it upregulated Foxp3^+^ regulatory T cells. Moreover, carboxyfluorescein diacetate succinimidyl ester-labeled UC-MSCs could be found in the lungs and kidneys after infusion [50]. Using NZB/W F1 mice, Chang et al. showed that human UC-MSC transplantation significantly delayed the onset of proteinuria, decreased anti-dsDNA, alleviated renal injury, and prolonged the life span [51]. Subsequent studies looking at the mechanisms showed that the treatment effect was not due to a direct engraftment and differentiation into renal tissue but rather to the inhibition of lymphocytes, the induced polarization of T helper 2 cytokines, and the inhibition of the synthesis of pro-inflammatory cytokines. Choi et al. showed that long-term repeated administration of human AT-MSCs ameliorated SLE in NZB/W F1 mice [52]. Compared with the control group, the AT-MSC-treated group had a higher survival rate, decreased histological and serological abnormalities, improved immunological function, and a decreased incidence of proteinuria. Transplantation of AT-MSCs led, on the one hand, to significant decreased levels of antibodies targeting dsDNA and blood urea nitrogen levels. On the other hand, it significantly increased serum levels of granulocyte-macrophage colony-stimulating factor, IL-4, and IL-10. A significant increase in the proportion of CD4^+^FoxP3^+^ cells with a marked restoration of their capacity to produce cytokines was observed in spleens from the AT-MSC-treated group.

### Animal models of systemic sclerosis

Among the various experimental models aiming at reproducing the SSc (genetic models, such as tight skin (TSK) Tsk1 and Tsk2 mice, UCD-200 chicken, Fra-2 mice, TGFβRIIΔκ, or inducible models using injections of bleomycin or vinyl chloride or graft-versus-host disease (GVHD) mice), none displayed exactly the three components of scleroderma in humans [53]. Indeed, two forms of SSc are defined in humans. The first one is characterized by extensive skin fibrosis (proximal and distal), common pulmonary fibrosis, and the presence of antibody directed against DNA topoisomerase 1. In regard to the second form, referred to as the “limited cutaneous” form, the skin disease is limited to the distal limbs and lung symptoms are rare. The autoantibodies detected in this second form are against centromere (the main target being the centromeric protein CENP-B) and not against DNA topoisomerase 1. The TSK mouse model is characterized mainly by skin lesions, which do not reach the dermis; others use the mismatch transplant BM or spleen cells in mice sublethally irradiated. A scleroderma-like syndrome associated with chronic GVHD was induced with skin and lung fibrosis and was associated with signs of autoimmunity. Finally, induction of fibrosis by bleomycin injection could be used. But none reproduced a true picture of scleroderma. The role of free radicals in the development of SSc was studied and this helped to develop a mouse model of scleroderma, based on repeated injection of hypochlorous acid [54]. This model mimics the diffuse form of the human disease (cutaneous sclerosis, pulmonary fibrosis, renal disease, and anti-topoisomerase antibodies) and is a more satisfactory way to test new therapeutic approaches than other models. Despite the lack of perfectly reproducible models of SSc, the effect of MSCs on fibrosis is known and has been studied in the model of fibrosis induced by bleomycin [55–57]. Injection of MSCs allowed investigators to limit the pro-inflammatory and pro-fibrotic bleomycin effect through a mechanism involving IL-1RA [58]. Even though this model only partially reproduces SSc disease, all of the in vitro and in vivo data suggest that MSCs may have a beneficial effect in SSc.

## Characteristics of mesenchymal stem cells derived from patients with systemic lupus erythematosus and systemic sclerosis

Because the majority of pathogenic autoreactive cells are the progeny of HSCs, it is conceivable that HSCs are involved in the AD process. BM-MSCs are key components of the hematopoietic microenvironment and provide support to hematopoiesis and modulate the immune system. Little is known about how MSCs are involved in immunological disorders. However, evidence has suggested that BM-MSCs from animal models and from patients with SLE and SSc exhibited impaired capacities of proliferation, differentiation, secretion of cytokines, and immune modulation. These alterations might be the consequence of the disease or play a fundamental role in the pathogenesis of SLE and SSc.

### Mesenchymal stem cells derived from patients with systemic lupus erythematosus

BM-MSCs from patients with SLE have impaired hematopoietic function [59] and show significantly decreased bone-forming capacity and impaired reconstruction of BM osteoblastic niche in vivo [43]. Moreover, BM-MSCs from patients with SLE seem larger and flatter in appearance during in vitro culture and grow progressively slower compared with those from controls, thus demonstrating early signs of senescence [60, 61]. This senescent state is associated with differences in gene expression profile of BM-MSCs between SLE patients and controls, resulting in abnormalities in actin cytoskeleton, cell cycling regulation, BMP/TGF-β, and MAPK signaling pathways in BM-MSCs from patients with SLE [62]. In their study, Gu et al. found that senescent BM-MSCs from patients with SLE display reduced ability to upregulate regulatory T cells [63]. An increased p16INK4A expression plays a major role in this cellular senescence process by regulating cytokine secretion as well as the ERK1/2 signaling pathway. Wnt/b-catenin signaling also plays a critical role in the senescence of SLE BM-MSCs through the p53/p21 pathway [64]. Finally, SLE BM-MSCs exhibit an increased apoptosis rate, as reflected by downregulation of Bcl-2 and upregulation of cytochrome C in the cytoplasm, and display an enhanced aging process as shown by the overproduction of intracellular reactive oxygen species, which might be linked with the upregulation of p-FoxO3 and its upstream gene AKT [65].

### Mesenchymal stem cells derived from patients with systemic sclerosis

Studies on BM-MSCs from patients with SSc are more limited. In patients with SSc, the osteogenic and adipogenic differentiation potentials of MSCs appear to be altered when they are isolated from the BM by direct selection of nerve growth factor receptor (CD271)-positive cells and not by the conventional technique of adhesion [66]. In these patients, the ability of MSCs to differentiate into endothelial progenitor cells appear reduced, and the endothelial progenitor cells obtained have a reduced ability to migrate and a lower pro-angiogenic potential [67]. Cipriani et al. showed that although BM-MSCs from SSc patients undergo premature senescence, they maintain considerable immunosuppressive functions and a normal ability to generate functional regulatory T cells [68]. In our study, we showed that the SSc BM-MSCs have fibroblast colony-forming units ability with a phenotype and a frequency similar to those of MSCs derived from healthy donors [69]. They differentiate into adipose and osteogenic cells with variabilities similar to those observed within the BM-MSCs from healthy controls. In regard to the immunoregulatory activity of MSCs in SSc, we reported that MSCs from patients were capable of supporting normal hematopoiesis and retained their immunosuppressive properties on T cells, thus confirming the data published by Bocelli-Tyndall et al. [69, 70]. We have recently shown a significant increase of the level of receptor type II TGF-β in MSCs from SSc patients compared with MSCs from healthy donors, associated with an activation of the TGF-β signaling pathway, leading to an increase in the synthesis of target genes, including the gene encoding collagen type 1 [71]. This activation of MSCs in response to stimulation by TGF-β, known for its major role in the pathogenesis of the disease, obviously limits their clinical use and justifies the use of allogeneic MSCs in these patients.

All of these findings suggest that BM-MSCs from patients with SLE or SSc are defective in regard to certain functions. Therefore, we can speculate that an allogeneic rather than an autologous MSC-based therapy might be preferable for treatment. Even though some data bring their early senescence to light, MSCs maintain some immunosuppressive properties that support the potential autologous clinical application. These data emphasize the necessity for a better understanding of the MSC involvement in the pathogenesis and the underlying MSC-immunomodulatory mechanisms.

## Hematopoietic stem cell-based and mesenchymal stem cell-based therapy in patients with systemic lupus erythematosus and systemic sclerosis

### Use of hematopoietic stem cell transplantation in systemic lupus erythematosus or systemic sclerosis

The use of HSCT in patients with AD to induce tolerance by resetting the immune responses is supported by both experimental data and clinical evidence. The direct relationship between the hematopoietic system and AD was evidenced in 1985 by Ikehara et al., who first demonstrated that AD originated from defects in the HSCs [72]. Thereafter, data from genetically prone and immunized animal models of AD treated with allogeneic, syngeneic, and autologous BM transplantation (BMT) showed that allogeneic BMT (but not syngeneic or autologous) could be used to treat AD-prone mice [73]. Conversely, the AD transfer was possible in normal mice after allograft from a mouse lupus nephritis showing that it was in fact a stem cell disorder. Consensus indications for the use of transplantation of BM-derived or peripheral HSCs to treat severe ADs were first elaborated in 1997 [74] and were updated in 2012 [75]. Today, more than 3500 patients worldwide have received an HSCT for an AD alone; approximately 200 autologous HSCTs were for refractory SLE and 500 were for severe SSc. This allowed a sustained and prolonged remission with qualitative immunological changes not seen with any other forms of treatment. In SLE, these beneficial effects were limited by the increased short-term mortality underlying the need to develop new strategies. In severe SSc, adequate prospective trials allowed investigators to ensure the safety of non-myeloablative autologous HSCT for SSc when careful patient selection, follow-up, and center effect are considered, to avoid misleading use of CY when it is unlikely to be clinically meaningfully effective. In case of allogeneic transplantation, more data suggest preclinical and clinical evidence for a graft versus autoimmunity effect in replacement of a dysfunctional immune system by allogeneic HSCT, which also represents an attractive prospect. In this setting, analysis of the regenerating adaptive immune system showed normalization of the restricted T-cell repertoire, with sustained shifts in T- and B-cell subpopulations from memory to naïve cell dominance supporting a thymic reprocessing and re-education of the reconstituting immune system [76, 77]. Disappearance of circulating plasmablasts and restoration of normal or raised levels of CD4+ and CD8 + FoxP3+ regulatory T cells were shown in SLE following autologous HSCT. This normalization was accompanied by complete inhibition of pathogenic T-cell response to autoepitopes from histones in nucleosomes [78, 79]. This has never been shown previously after the use of conventional immunosuppressive therapies. Such clinical and immunological results allowed investigators to take into account the non-specific immunosuppressive changes, which can be observed both in blood and in tissues after cytotoxic therapy [76, 80], and immune re-educative changes supporting immune tolerance [81]. Therefore, for the first time in AD treatment, the interruption of the vicious circle of autoimmunity allowed the emergence of normal regulatory mechanisms and the eradication of the last auto-reactive T cell, which is one of the proposed mechanisms for using HSCs in the treatment of SLE and SSc.

### Mesenchymal stem cell-based therapy in systemic lupus erythematosus and systemic sclerosis

Discovery and identification of MSCs within the BM content and of their therapeutic properties have led us and others to use MSCs derived from various tissues to treat AD. Indeed, the supportive function for HSCs in the BM niche and the immunomodulatory capacities of MSCs suggest their potential use for cell therapy. Allogeneic donor-derived BM-MSCs have already been used in several phase I and II and very few phase III clinical trials for the treatment of acute GVHD following allogeneic HSCT for leukemia or hematological malignancies [82]. With a better understanding of the combined components of autoimmunity and autoinflammmation in each AD, there is a rationale to propose combined therapies with different tools.

BM-MSCs and UC-MSCs have been transplanted in patients with severe SLE, who were not responsive to conventional therapies. The 4-year follow-up demonstrated that about 50 % of the patients entered clinical remission after transplantation, although 23 % of the patients relapsed [83]. MSC infusion induced disease remission for lupus nephritis [84], diffuse alveolar hemorrhage [85], and refractory cytopenia [86]. The multi-center clinical study showed that 32.5 % of patients achieved major clinical response (13 out of 40) and 27.5 % of patients achieved partial clinical response (11 out of 40) during a 12-month follow-up, respectively. However, 7 (17.5 %) out of 40 patients experienced a disease relapse after 6 months of follow-up, after a prior clinical response, which indicated that another MSC infusion would be necessary after 6 months [87].

Few data are available about MSC-based therapy in patients with SSc. A patient with severe refractory SSc received an intravenous injection of allogeneic MSCs [88]. Three months after injection of MSCs, a significant decrease in the number of digital ulcers was observed. At 6 months, blood flow to the hands and fingers seemed significantly improved, and transcutaneous partial pressure of oxygen was increased. Rodnan skin score dropped from 25 to 11. The titer of anti-Scl-70 antibody, however, remained high, and enumeration of lymphocytes T, B, and natural killer cells did not change. This first observations were supplemented by four other cases reported by the same German team using allogeneic MSCs to treat severe forms of SSc, without major side effects or specific abnormalities observed after respective follow-ups of 44, 24, 6, 23, and 18 months [89]. The first two patients received fresh MSCs, whereas the three others received cryopreserved allogeneic MSCs. No conclusion about the efficacy of the MSC transplantation can be drawn from these clinical cases, although skin improvement was noted in three out of five cases and these patients did not have a detailed immunomonitoring.

Although further studies are necessary, preclinical and clinical data underline the therapeutic potential of MSCs in patients with SLE and SSc. Now it is important to design a controlled study to further investigate the clinical efficacy of MSC transplantation, compared with conventional immunosuppressive therapies, or the efficacy of MSC transplantation combined with immunosuppressive drug treatment compared with drugs alone. Careful patient selection and performance are crucial for the proper use of this therapy.

## Note

This article is part of a thematic series on Biology and clinical applications of stem cells for autoimmune and musculoskeletal disorders, edited by Christian Jorgensen and Anthony Hollander. Other articles in this series can be found at http://www.biomedcentral.com/series/MSC

## Abbreviations

AD: Autoimmune disease
AT: Adipose tissue
BAFF: B-cell-activating factor of the tumor necrosis factor family
BM: Bone marrow
BM-MSC: Bone marrow-derived mesenchymal stem cell
BMT: Bone marrow transplantation
CY: Cyclophosphamide
DC: Dendritic cell
dsDNA: Double-stranded DNA
GVHD: Graft-versus-host disease
HSC: Hematopoietic stem cell
HSCT: Hematopoietic stem cell transplantation
IFN-γ: Interferon-gamma
IL: Interleukin
ISCT: International Society for Cellular Therapy
MHC: Major histocompatibility complex
MSC: Mesenchymal stem cell
SLE: Systemic lupus erythematosus
SSc: Systemic sclerosis
TGF-β: Transforming growth factor-beta
TSK: Tight skin
UC: Umbilical cord
UC-MSC: Umbilical cord-derived mesenchymal stem cell
